# 

**Published:** 1914-02-07

**Authors:** 


### CONTENTS.

| | PAGE |
|---|---|
| **Hospital Staff Appointments** ... ... ... | 493 |
| **South Shields Guardians withdraw their Resolution** ... ... ... ... ... ... | 494 |
| **Hospital and Institutional News** ... ... ... | 495 |

An Institute of Pathology at the Middlesex Hospital—Mental Hospitals and Early Cases—The Psychiatric Clinic and the Voluntary System—Leanchoil Hospital and Lord Strathcona—A Hospital and Party Politics—Staff Changes at Claybury Hospital—A Discussion on Sanatoria Construction—Lord Strathcona's Hospital Bequests—The Registration of Still-births—Queen Alexandra and St. Katharine's Hospital—St. George's Hospital Special Court—Salaries of Tuberculosis Officers—The Single Pavilion: A Forecast—New Poor-Law Hospital at Newcastle—Mr. Holland Succeeds to the Peerage—Alleged Negligence of an Anæsthetist—New Premises at Norwich—Factory Lectures on Venereal Disease—Red Cross Aeroplanes—Staff Changes at Brentford Infirmary—The Machinery of the County Council—The Hospital for Poor Jews at Jerusalem—A Cottage Hospital's Litigation—The New Glasgow Children's Hospital—A Notable Cottage Hospital Treasurer—The Growth of the German Hospital—Public Pharmacists' Association's New Chairman—This Week's Drug Market.

| | PAGE |
|---|---|
| **Modern Methods Series.—II.** ... ... ... ... | 501 |
| Artificial Pneumothorax—Treatment of Aneurysm—Diathermy. | |
| **Women and War** ... ... ... ... ... ... | 502 |
| The Convoy Corps in the Balkans. | |
| **The Health Temples of Ancient Greece** ... | 503 |
| **The School Doctor's Problems** ... ... ... | 505 |
| Flat Foot in School Children. | |
| **Some Impressions of Continental Hospitals** ... | 506 |
| By a Travelling Resident Medical Officer. | |
| **Put St. George's Hospital First** ... ... ... | 507 |
| The Past, the Present, and the Future. | |

[*See over.*]

### CONTENTS—(*continued*).

| | PAGE |
|---|---|
| **Reports on Hospitals of the United Kingdom—Series III.** ... | 509 |
| By Sir HENRY BURDETT, K.C.B., K.C.V.O. Bedford County Hospital. | |
| **Hospital Architecture and Construction** ... | 510 |
| New Units at the Royal Infirmary, Sheffield. | |
| **National Insurance Act Developments** ... | 511 |
| The New Regulations as to Arrears of Contributions. | |
| **Public Pharmacists' Association** ... | 512 |
| **Doctor's Wills** ... | 512 |
| **The Institutional Library** ... | 513 |
| The Latest Hospital History. Radium Therapy. A Modern "De Senectute." Sanatorium Lectures. | |
| **Round the World** ... | 515 |
| Fellow-Workers and Their Doings. | |
| **The Editor's Letter-Box** ... | 516 |
| North Staffordshire Infirmary. Treatment in Mental Hospitals. Instruction in Psycho Therapy. A Warning to Non-Panel Men. | |
| **The Profession of Medicine** ... | 518 |
| The National Medical Union. State Endowment of Unqualified Practice. | |
| **The Institutional Worker** ... (Suppl.) | 1 |
| Contracts for Supplies. | |
| **Our Bureau of Information** ... „ | 2 |
| Employment and Training. Questions for February. The Sick and in Need. | |

